# After the Open Dialogue: a Within-Area Comparison of Treatment, Experiences, and Outcomes in Western Lapland 20 Years after Service Fragmentation

**DOI:** 10.1007/s10597-026-01591-z

**Published:** 2026-01-22

**Authors:** Tomi Bergström, Jouko Miettunen, Katja Partanen, Vilhelmiina Yrjänheikki, Katariina Rantamaa, Teija Hietasaari, Enni Öfverberg, Tapio Gauffin, Jouni Petäjäniemi

**Affiliations:** 1 University of Jyväskylä, Jyvaskyla, Finland; 2 University of Oulu, Oulu, Finland; 3 Oulu University Hospital, Oulu, Finland; 4 Wellbeing Services County of Lapland, Kemi, Finland; 5 University of Helsinki, Helsinki, Finland

**Keywords:** Natural experiment, Service integration, Dialogical practice, Health service governance

## Abstract

This within-region service comparison examines the long-term sustainability and outcomes of the Open Dialogue (OD) approach in routine psychiatric service in the Länsi-Pohja Hospital District, Finland (Western Lapland catchment area). Originating in the 1980s, OD emphasizes immediate response, social network involvement, continuity of care, and dialogical practice. While OD was implemented district-wide by 1990, the municipalization of outpatient services in the late 1990s led to fragmentation. This study compares treatment processes, user experiences, and outcomes between services that remained under hospital district governance and a previously municipalized unit recently reintegrated into the district. Data were drawn from the PSILEAPS project, including surveys of 117 service users and their staff conducted between 2022 and 2023. Follow-up data was collected in 2024 by reviewing case records to determine whether service users remained in treatment 1–2 years after the survey and whether treatment ended by mutual agreement. Descriptive analyses used inverse probability weighting to account for baseline differences. Findings indicate that hospital district–run units delivered more OD consistent care, with greater family involvement and use of home and network meetings. In hospital district-group these elements were rated more positively by users and staff, and treatment in this group was associated with mutually agreed treatment endings at follow-up. Despite shared origins and similar intake systems, structural differences associated with the development of distinct treatment cultures and outcome patterns. Beyond structural reforms and access to services, maintaining the core principles of OD may depend on organizational stability and sustained support for a certain treatment culture.

## Introduction

Open Dialogue (OD) is a network-oriented approach to mental health care that integrates both service organization and a dialogical method for clinical practice (Razzaque & Stockmann, 2016). It emerged through bottom-up service development in the southwestern Finnish Lapland. In the early 1980 s, clinicians at the local psychiatric facility, Keropudas Hospital, began applying psychoanalytically informed psychotherapy and systemic family therapy in the treatment of psychosis. However, these predefined therapeutic frameworks were soon found to be too rigid and objectifying to respond effectively to the diverse and complex needs of individuals referred to public psychiatric services (Seikkula & Olson, 2003).

Influenced by the need-adapted approach (Alanen, 2009)—which emphasizes tailoring care to the evolving needs of individuals and their social networks—staff began organizing network treatment meetings involving all those affected by a given crisis. Drawing on collaborative (Anderson & Goolishian, 1988) and reflective therapeutic traditions (Andersen, 1987), these meetings avoided predetermined agendas and interventions. Instead, they focused on amplifying the voices of all participants. This shift encouraged professionals to move away from expert-driven models towards polyphonic dialogues that seemed to improve outcomes (Seikkula & Olson, 2003); the dialogical stance not only helped generate new meanings and shared understanding around difficult situations, but also reduced psychological distress by legitimizing diverse perspectives. Rather than pathologizing or defending experiences, participants engaged in joint reflection within their social contexts.

The orientation toward shared meaning-making allowed for a more nuanced understanding of mental health crises and enabled care to be flexibly tailored on a case-by-case basis. These developments led staff at Keropudas Hospital to formalize a region-wide model that promoted dialogism in all clinical encounters (Seikkula & Olson, 2003). This effort was supported by the Länsi-Pohja Hospital District, established in the late 1980 s, which brought the region’s mental health units under the same organizational structure (Haarakangas, 1997).

By the mid-1990s, new region-wide system had become well established, guided by seven core principles that came to define Open Dialogue approach (Seikkula et al., 2011): (1) Immediate Help, ensuring a response within 24 h if needed; (2) a Social Network Perspective, involving those affected by the crisis; (3) Flexibility and Mobility, adapting to individual needs; (4) Responsibility, with the initial team maintaining involvement and facilitation of network-treatment meetings; (5) Psychological Continuity, with consistent care relationships with the same team members; (6) Tolerance of Uncertainty, avoiding rapid interpretations and decisions, allowing thus space for dialogue and meaning to emerge; and (7) Dialogism emphasizes open, co-constructed understanding through the polyphony of multiple voices, where meaning emerges from interaction rather than being imposed by any single perspective.

Research, including quasi-experimental (Seikkula et al., 2003) and historical comparisons (Seikkula et al., 2006, 2011), demonstrated positive outcomes of OD-based services in treating first-episode psychosis, particularly in terms of symptom reduction and social functioning. Subsequent long-term, register-based cohort follow-ups with nationwide comparisons have confirmed the stability of these outcomes (Bergström et al., 2018).

Despite promising results, national health care reforms in the late 1990 s led to the municipalization of most outpatient clinics, fragmenting the previously unified service system. To preserve dialogical practices, many experienced professionals moved to newly established adolescent services that continued OD-based care, ensuring early network-oriented responses across organizational boundaries (Bergström et al., 2022a). In contrast, adult services became increasingly fragmented, with only the Tornio outpatient clinic and Keropudas Hospital remaining under the hospital district. This shift led to discrepancies in training, supervision, and resource allocation, especially within municipalized units. Consequently, while some units continued to systematically operate under the OD organizational framework, others developed their own treatment cultures and approaches.

The potential within-region treatment culture variations in the Länsi-Pohja hospital district offers a unique naturalistic experiment to assess the sustainability and impact of dialogical practices in real-world service settings. As international interest in dialogical approaches continues to grow (Pocobello et al., 2023), these insights are important for understanding the conditions that support the long-term sustainability of Open Dialogue within real-world mental health systems.

The primary aim of this naturalistic, within-region comparison was to examine whether long-standing organizational differences between clinics within the Open Dialogue catchment area were reflected in variations in treatment practices, treatment experiences, and outcomes. More specific questions were as follows:

1. How do treatment practices differ between the Open Dialogue–based hospital district units and the municipal unit?
2. How do service users and staff experience and rate the helpfulness of treatment, and do these experiences differ across units?
3. Do treatment outcomes differ between the units, particularly regarding improvement in well-being and the longer-term need for treatment?

## Methods

### Setting

To address the primary aim of this study, we conducted a post hoc analysis of data from the PSILEAPS project, which was designed to survey mental health services in the Länsi-Pohja Hospital District in 2022 (Bergström et al., 2025). The original aim of the PSILEAPS project was to explore whether routine clinical data could be systematically collected for service development by integrating elements of participatory design into everyday clinical practice. Although the project was not originally designed for between-unit comparisons, the breadth of routine data gathered across settings provides a unique opportunity to examine variations in treatment practices, treatment experiences, and outcomes within a real-world service system.

At the time of data collection in 2022, the Länsi-Pohja Hospital District encompassed two towns (Kemi and Tornio) each with a population of approximately 20,000, along with 4 smaller surrounding municipalities. The region was served by six adult outpatient clinics (located in Tornio, Kemi, Simo, Keminmaa, Tervola, and Ylitornio), each primarily responsible for providing mental health services to their local residents. In addition, one psychiatric inpatient facility—Keropudas Hospital, located in Tornio—provided inpatient care and acute outpatient care by managing the low-threshold service intake for the entire region.

All services started to operate under the same organizational structure from the establishment of the hospital district in the late 1980 s onward. As a result, the traditional divide between primary and specialized mental health care was removed, and access to services was centralized through the Keropudas Hospital outpatient clinic (Haarakangas, 1997). Service intake was negotiated collaboratively, determining when and where meetings would take place and who should be involved. In acute situations—such as suicidal ideation or psychosis—a network meeting was convened within 24 h, typically in the person’s home or another preferred location.

To uphold the dialogical ethos in meetings, clinicians embraced a “not-knowing” stance, intentionally refraining from making premature clinical judgments or applying predetermined interventions. Instead, they prioritized open-ended questioning, active listening, and shared reflection as core practices. Treatment planning and meaning-making took place collaboratively during meetings, with additional sessions scheduled promptly when consensus was not reached or when re-adjustments to the treatment process were needed. Recognizing the importance of trust, continuity, and safety, practitioners worked in consistent pairs and followed cases over time. This required continuation of care over municipal boundaries, adequate staffing, regular supervision, and ongoing training to support a reflective and non-authoritarian approach. To ensure sustained resources and continuity, a three-year in-house training program was offered for all mental health professionals in region.

By the mid-1990s, all units in the region were operating within a unified Open Dialogue–based service system, and approximately 90% of the mental health workforce had received the three-year, on-the-job Open Dialogue training. However, toward the late 1990 s, national service reforms led to the municipalization of most outpatient services. Based on local political decision-making, in Länsi-Pohja only the Tornio outpatient clinic, Child and adolescent clinic and Keropudas hospital continued to operate within the hospital district, while the Kemi outpatient clinic and all smaller outpatient units were transferred to municipal governance and began functioning under different organizational frameworks.

Two years prior to the PSILEAPS project, the Kemi outpatient clinic was reintegrated into the hospital district after having operated under municipal governance over two decades. Outpatient services in the smaller municipalities, however, continued to function independently under local governance. Despite this fragmentation, intake across the region was centrally coordinated through the Keropudas outpatient clinic, which operated a 24/7 emergency phone line and accepted walk-ins without referral. Based on clinical urgency, hospital staff either initiated first treatment meeting themselves or referred individuals to local outpatient clinics, based on their place of residence.

Although municipalities favored local referrals due to reimbursement structures, in more severe cases Keropudas staff were authorized to coordinate care across municipal boundaries, either collaboratively or independently of municipalized services. In parallel, municipalities maintained their own intake and referral procedures. Importantly, no specialized or diagnosis-specific services existed in the region; all outpatient clinics provided the full range of general adult mental health care for their local population, which contributed to a broadly similar case mix across units. The primary exception was the Keropudas outpatient clinic, whose broader clinical mandate encompassed more complex presentations and consequently involved somewhat more severe cases.

This organizational configuration is relevant for the present naturalistic comparison: although the clinics had operated for years under different administrative arrangements, they served broadly similar populations. This reduces the likelihood of strong confounding by indication and facilitates an examination of how long-term organizational differences may be reflected in treatment practices, care experiences, and outcomes.

### Participants and Measures

During the original PSILEAPS project, data were collected in designated two-week periods during which all individuals involved in mental health services within the hospital district were invited to participate. Participants completed structured questionnaires and allowed the use of routine clinical data for baseline and follow-up information. The questionnaire, developed as a part of the project through a participatory process involving staff, service users, and peer experts, gathered information on treatments received, their perceived helpfulness, and changes in the individual’s situation over the previous month. With consent, staff and social network members also completed corresponding questionnaires to provide parallel perspectives.

In 2022, 89 service users participated (approximate response rate 50%). Recruitment resumed in 2023 to reach the target sample, resulting in a final sample of 117 participants (79% of target). Due to a declining response rate and major national health-care reforms, data collection was not extended further. The sample overrepresented individuals with mood disorders, women, and those with longer treatment histories. Detailed information on sampling procedures and instruments is available elsewhere (Bergström et al., 2022b, 2025).

Participants were recruited from units administered by the hospital district at the time: the Kemi and Tornio outpatient clinics and Keropudas Hospital. Most therefore resided in Tornio or Kemi, though some individuals from surrounding municipalities were included if they received care through district services. Each of the outpatient clinics typically employed 6–10 nurses, 3–6 specialized professionals (e.g., psychologists, occupational therapists, social workers), and 1–2 physicians, with staffing levels varying according to workforce availability.

Staff members completed one questionnaire for each service user whose treatment they were involved in during the data-collection period. Thus, an individual worker could provide multiple responses if they were responsible for several service users, and staff questionnaires correspond directly to the same treatment processes for which the service users themselves provided data. Accordingly, the number of worker responses reflects the number of evaluated treatment processes rather than the number of unique staff participants.

For this sub-study, treatment processes were classified into two groups:

1. Hospital district–operated units (research group): processes in which service users received care primarily from the Tornio outpatient clinic and/or Keropudas outpatient services.
2. Municipalized unit (comparison group): processes in which service users received care primarily from the Kemi outpatient clinic, which had operated under municipal administration until its recent reintegration.

The PSILEAPS questionnaires assessed the perceived helpfulness of different treatment modalities using a visual analog scale (0–10; 0 = strongly disagree, 10 = strongly agree), the extent to which treatment aligned with participants’ expectations, and perceived changes in psychological well-being (0–10; 0 = extreme decline, 10 = extreme improvement). Staff also rated whether they could work according to their preferred clinical approach. Demographic and clinical data were drawn from self-report and case records, and staff completed the Brief Psychiatric Rating Scale (BPRS) (Overall & Gorman, 1962) to evaluate severity of current symptoms.

Table 1 summarizes the data sources and questionnaire items included in this analysis. Note, that social network-member responses were excluded from this sub-study because their markedly lower participation rate created a strong selection bias and would have reduced the clarity and interpretability of the reported results (Bergström et al., 2025). These data will be analyzed separately in a subsequent phase of the research.

**Table 1:** Overview of data collected from respondent groups

Respondent group	Demographic information^a^	Clinical information^a^	Questions used in this study^b^
Service users	• Age • Gender • Education • Residence • Civil status • Number of children • Employment status • Sickness leave • Disability allowance	• Diagnoses (ICD-10) • BPRS score • Time to first meeting • Changes in treatment team members • Ongoing rehabilitation psychotherapy • Overall duration of mental health treatment • Number of readmissions • Duration of hospital treatment • Psychotropic medication history (past, ongoing, type, dose, off-label •Treatment status at the follow-up	**Helpfulness of treatment elements (0 = strongly disagree**,** 10 = strongly agree)** • Continuity of care • Rehabilitation psychotherapy • Pair work • Psychiatric hospital care • Supported housing • Embodiment exercise • Home visits • Occupational therapy • Psychotropics • Social work • Peer support • Family engagement • Professional networks • Whether treatment followed the client’s treatment preferences **Well-being (0 = extreme decline**,** 10 = extreme improvement)** • Change in psychological well-being in past month
Care team members	• Work experience • Profession • Work unit • In-service training • OD training	Not applicable	• Corresponding questions on the service user’s situation

^a^Directly and/or case notes if applicable

^b^Sections and questions used in this study; a full description of the dataset is provided in (Bergström et al., 2022)

Participants provided informed consent for linkage to national health registers to assess long-term outcomes, conditional on future funding. However, due to lack of funding at the time of this sub-analysis, primary outcomes were derived directly from case notes reviewed in 2024. These records were used to determine whether treatment had concluded within 1–2 years of recruitment (depending on the time of inclusion), and whether it ended by mutual agreement—indicating that both the service user and care team agreed there was no longer a need for services—or due to dropout, or continuation with psychotropic medication management only.

To address the main aim of this study, the extensive data collected in the original project were reorganized into four analytic domains reflecting the conceptual sequence from service context to user experiences and outcomes:

1. Baseline characteristics
  For service users, demographic variables included age, gender, family status, education, and employment status. Clinical variables included work capability, prior treatments, diagnoses, and BPRS scores at recruitment. For staff members, information on profession, work unit, years of work experience, and training (including OD training) was used.
2. Treatment characteristics
  This domain captured whether the service user received treatment modality in question during their care.
3. Treatment experiences
  Service users and staff rated the perceived helpfulness of each treatment modality they received or delivered on a scale from 0 to 10 (0 = not helpful, 10 = extremely helpful).
4. Treatment outcomes
  Short-term outcomes were assessed using service users’ self-rated change in well-being during the month preceding participation (0 = extreme decline, 10 = extreme improvement).

Long-term outcomes were assessed using case records to determine treatment status at follow-up (1–2 years after recruitment). Treatment status was categorized as: continued treatment, psychotropic medication management only, treatment ended by mutual agreement.

### Statistical Analysis

Given the non-randomized, post hoc design of the study and the potential for selection bias, baseline differences were addressed using stabilized inverse probability of treatment weighting (SIPTW) (Austin & Stuart, 2015). SIPTW creates a weighted sample in which the distribution of baseline characteristics is balanced between groups, approximating the conditions of a randomized study. This reduces the influence of selection bias and allows group comparisons to reflect differences more closely attributable to the treatment context rather than underlying differences in service user characteristics.

First, logistic regression was used to estimate the probability of assignment to research group vs. comparison group, based on covariates including sex, education, employment and family status, diagnosis, work capability, BPRS score, treatment duration, and follow-up time. Stabilized IPTW values were then calculated and applied to balance differences across groups. Descriptive statistics, chi-square tests, and independent samples t-tests were then used to compare treatment characteristics, treatment experiences, and outcomes across the two groups.

Weighted logistic regression was used to evaluate whether group assignment predicted the likelihood of treatment ending through mutual agreement. This was considered a positive indicator of treatment outcome, as it reflected a shared evaluation by both staff and the service user that the situation had improved sufficiently and that continued mental health treatment was no longer necessary.

A p-value of < 0.05 was considered statistically significant; however, given the exploratory nature of the study, the findings should be interpreted as hypothesis-generating rather than confirmatory and corrections for multiple comparisons were not applied.

#### Ethical Considerations

The PSILEAPS study protocol was reviewed and approved by *the North Ostrobothnia Hospital District Ethics Committee.* All participants provided written informed consent. It was emphasized that participation or non-participation—would not affect the care received. Similarly, staff members gave written consent with assurance that participation would not influence their employment or clinical duties.

### Results

#### Baseline Characteristics of Service Users

At the time of recruitment, service users in the research group were more likely to be male, less likely to be employed or in school, and less likely to be in a relationship (Table 2). They also had on average a longer duration of treatment compared to the comparison group. However, none of these differences reached statistical significance.

**Table 2:** Baseline characteristics of service users prior and after weighting

	Non-weighted			SIPT-weighted		
	Research group *n* = 58	Comparison group *n* = 59	*p*	Research group *n* = 58	Comparison group *n* = 59	*p*
Age, mean (SD)	38 (14)	38 (15)	0.4	38 (15)	38 (15)	0.4
Gender, Female	40 (69%)	47 (80%)	0.1	44 (76%)	44 (75%)	0.9
Higher education	8 (14%)	9 (15%)	0.8	8 (14%)	8 (14%)	0.9
In working or school	28 (48%)	35 (59%)	0.4	30 (53%)	31 (53%)	0.9
Disability pension	21 (36%)	25 (42%)	0.5	21 (37%)	21 (36%)	0.9
In relationship	27 (47%)	37 (63%)	0.1	31 (54%)	31 (53%)	0.9
Children	27 (47%)	25 (42%)	0.9	25 (43%)	26 (44%)	0.9
Treatment length over a year	41 (71%)	35 (59%)	0.1	38 (65%)	37 (63%)	0.8
Diagnosis F1 F2 F3 F4 F5 F6 F7 F8 F9	3 (5%) 4 (7%) 34 (58%) 22 (37%) 2 (3%) 8 (14%) 0 2 (3%) 5 (9%)	3 (5%) 6 (10%) 35 (59%) 22 (37%) 0 7 (12%) 1 (2%) 0 4 (7%)	0.8 0.5 0.9 0.9 0.2 0.7 0.4 0.1 0.8	3 (5%) 5 (9%) 34 (60%) 22 (38%) 1 (2%) 9 (15%) 0 1 (2%) 5 (8%)	3 (5%) 5 (9%) 36 (61%) 22 (37%) 0 6 (11%) 1 (2%) 0 3 (5%)	0.8 0.9 0.9 0.9 0.4 0.4 0.3 0.3 0.5
BPRS-18, mean (SD)	35 (11)	36 (10)	0.1	35 (10)	35 (10)	0.4
Follow-up time two-years	46 (79%)	47 (78%)	0.9	45 (79%)	45 (78%)	0.9

*SD* Standard deviation

Subgroup analysis indicated that these differences were largely attributable to the unique service user profile at the Keropudas outpatient clinic, as anticipated given the clinic’s clinical mandate. Service users recruited through Keropudas were more likely to have been in treatment for over a year (84%, compared to 60% in other units), to have lower levels of education (12% with higher education vs. 15% in other units), to be work-disabled (45% vs. 30% in Tornio and 42% in Kemi), to be male (40% vs. 20%), and to be unemployed (55% vs. 44%).

After applying SIPTW, no statistically significant differences remained between the groups in baseline demographic or clinical characteristics.

#### Baseline Characteristics of Workers

A total of 54 workers participated in the study, with 36 employed in units assigned to the research group and 18 in the comparison group. Among the respondents, 25 (70%) in the research group and 13 (72%) in the comparison group were nurses. The remaining participants were specialized professionals and doctors. A significantly higher proportion of workers in the research group had completed the three-year on-the-job Open Dialogue training (26 [72%] vs. 5 [28%], *p* < 0.05). On average, workers in the research group also had significantly more work experience (17 years vs. 10 years, *p* < 0.05), reflecting the higher staff turnover in the comparison group.

#### Treatment Characteristics

Following weighting for baseline variables, several statistically significant differences in treatment characteristics were observed between the groups (Table 3). Service users in the research group were significantly more likely to receive care from staff with OD training (83% vs. 56%, *p* < 0.05) and families were more often involved in the treatment (62% vs. 39%, *p* < 0.05). Home visits were also more common in the research group (17% vs. 5%, *p* < 0.05), as was access to occupational therapy (29% vs. 6%, *p* < 0.05) and group treatment (26% vs. 12%, *p* < 0.05).

Network treatment meetings involving other professionals were more frequently reported in the research group (41% vs. 27%), though this difference was not statistically significant. Other forms of support, such as embodied exercises (e.g. mindfulness, relaxations), social work, and peer support, were also more commonly reported in the research group but did not reach statistical significance. In both groups, the first treatment meeting was arranged within weeks of the initial contact. There was no significant difference in the use of psychotropic medication between the groups.

**Table 3:** Treatment characteristics

	Non-weighted			SIPT-weighted		
	Research group, *n* = 58	Comparison group, *n* = 59	*p*	Research group, *n* = 58	Comparison group, *n* = 59	*p*
OD-trained worker	48 (83%)	33 (56%)	0.002	42 (81%)	33 (52%)	0.001
Start of treatment < 1 month	55 (95%)	55 (93%)	0.7	55 (95%)	55 (93%)	0.7
Continuity (same workers)	51 (88%)	51 (86%)	0.8	50 (86%)	51 (86%)	0.9
Family engagement	34 (57%)	25 (42%)	0.08	36 (62%)	23 (39%)	0.01
Home visits	10 (17%)	3 (5%)	0.04	10 (17%)	3 (5%)	0.04
Pair work	54 (91%)	48 (83%)	0.2	53 (91%)	47 (83%)	0.2
Professional networks	24 (41%)	16 (27%)	0.1	24 (41%)	16 (27%)	0.1
Rehabilitation psychotherapy	4 (7%)	7 (12%)	0.4	4 (7%)	6 (10%)	0.5
Hospital treatment	14 (24%)	12 (20%)	0.6	15 (26%)	12 (21%)	0.5
Embodiment exercises	11 (19%)	9 (15%)	0.6	10 (17%)	7 (12%)	0.4
Occupational therapy	16 (28%)	6 (10%)	0.02	17 (29%)	6 (10%)	0.01
Psychotropics	48 (82%)	45 (76%)	0.4	46 (79%)	44 (76%)	0.6
Social work	31 (53%)	22 (37%)	0.08	30 (53%)	24 (41%)	0.2
Peer support	9 (16%)	9 (15%)	0.9	8 (14%)	11 (19%)	0.4
Group treatment	14 (24%)	8 (13%)	0.1	15 (26%)	7 (12%)	0.04

#### Treatment Experiences

Across both groups, continuity of care—defined as being treated consistently by the same professionals—was the most highly valued component of treatment (Table 4). However, on average it was rated significantly more positively by service users in the research group (*p* < 0.05).

**Table 4:** Service users’ experiences of the helpfulness of different treatments

	Non-weighted			SIPT-weighted		
	Research group, *n* = 58	Comparison group, *n* = 59		Research group, *n* = 58	Comparison group, *n* = 59	
	Mean^a^ (SD)	Mean^a^ (SD)	p	Mean^a^ (SD)	Mean^a^ (SD)	p
Continuity (same workers)	9 (1)	8 (1)	0.004	9 (1)	8 (2)	0.003
Family engagement	8 (2)	5 (3)	< 0.001	8 (2)	4 (3)	< 0.001
Home visits	7 (3)	4 (3)	0.1	7 (3)	4 (4)	0.1
Pair work	7 (3)	8 (2)	0.2	7 (3)	7 (3)	0.2
Professional networks	7 (3)	5 (3)	0.04	7 (2)	5 (3)	0.04
Rehabilitation psychotherapy	6 (2)	7 (3)	0.5	6 (2)	6 (4)	0.4
Hospital treatment	6 (3)	7 (3)	0.2	6 (3)	7 (3)	0.2
Embodiment exercises	7 (3)	6 (3)	0.3	7 (3)	6 (4)	0.3
Occupational therapy	7 (3)	8 (3)	0.2	8 (3)	8 (3)	0.3
Psychotropics	7 (3)	8 (2)	0.4	7 (3)	8 (2)	0.4
Social work	7 (3)	6 (3)	0.1	7 (3)	6 (3)	0.1
Peer support	7 (3)	6 (3)	0.9	7 (3)	7 (3)	0.3
Group treatment	7 (3)	7 (3)	0.4	7 (2)	8 (3)	0.4

^a^0=Not helpful, 10 = Extreme helpful

*SD* standard deviation

Family involvement was also rated more favourably in the research group, with an average helpfulness score of 8 out of 10, compared to less than 5 out of 10 in the comparison group, where it was generally considered unhelpful. Home visits and network treatment meetings involving multiple professionals were also rated as more helpful in the research group (average score of 7), though these differences did not reach statistical significance.

Other treatment elements, such as pair work, occupational therapy, group treatment, peer support, and psychotropics, were rated similarly favourably across both groups. Interventions such as embodied exercises, rehabilitation psychotherapy, and hospital treatment were generally rated more neutrally in both groups (average score around 6).

Staff responses closely reflected service user feedback (Table 5). Continuity of care was highly valued in both groups but received significantly higher ratings in the research group. Staff in the research group also reported significantly greater satisfaction with family involvement and interprofessional collaboration during network treatment meetings, though appreciation of these elements were high in both groups.

**Table 5:** Workers’ experiences of the helpfulness of different treatments

	Non-weighted			SIPT-weighted		
	Research group, *n* = 58	Comparison group, *n* = 59		Research group, *n* = 58	Comparison group, *n* = 59	
	Mean^a^ (SD)	Mean^a^ (SD)	p	Mean^a^ (SD)	Mean^a^ (SD)	p
Continuity (same workers)	9 (1)	8 (1)	0.007	9 (1)	8 (1)	0.003
Family engagement	8 (2)	7 (1)	0.04	8 (2)	7 (1)	0.04
Home visits	8 (1)	7 (1)	0.2	8 (1)	7 (1)	0.2
Pair work	8 (3)	8 (2)	0.1	8 (2)	7 (1)	0.1
Professional networks	8 (2)	7 (2)	0.04	8 (1)	7 (2)	0.04
Rehabilitation psychotherapy	7 (2)	8 (3)	0.3	7 (2)	8 (3)	0.4
Hospital treatment	4 (2)	6 (2)	0.08	5 (3)	6 (2)	0.1
Embodiment exercises	8 (2)	8 (1)	0.4	7 (2)	8 (1)	0.3
Occupational therapy	9 (1)	8 (1)	0.1	9 (1)	8 (1)	0.05
Psychotropics	7 (3)	8 (2)	0.4	7 (2)	8 (2)	0.4
Social work	8 (2)	7 (2)	0.02	8 (2)	7 (2)	0.04
Peer support	8 (1)	8 (1)	0.4	8 (1)	8 (2)	0.4
Group treatment	7 (2)	8 (1)	0.02	7 (2)	8 (1)	0.04

If there was more than one worker’s response regarding the same treatment process, the figure represents the mean of those responses

*SD* standard deviation

^a^0=Not helpful, 10 = Extreme helpful

In both groups, hospital treatment was rated less positively by staff compared to service users. Other treatment components were evaluated similarly across groups by staff respondents.

#### Outcomes

Service users in the research group reported greater improvement in their condition during the month preceding recruitment (average score 5.9 vs. 4.9 on a scale from 0 = severe deterioration to 10 = strong improvement) (Table 6). Overall satisfaction with treatment was high in both groups. Service users in the research group were more likely to report that staff worked in a manner they considered helpful, and staff themselves also reported being able to work in alignment with their preferred way of working. However, only the difference in staff reports reached statistical significance.

**Table 6:** Outcome

	Non-Weighted			SIPT-weighted		
	Research group, *n* = 58	Comparison group, *n* = 59	*p*	Research group, *n* = 58	Comparison group, *n* = 59	*p*
Change in well-being over the past month, 0 = severe decline, 10 = extreme improvement (mean (SD))	5.8 (2.5)	4.8 (2.2)	0.01	5.9 (2.4)	4.9 (2)	0.01
Ongoing treatment contact at follow-up	35 (60%)	42 (71%)	0.2	35 (60%)	41 (71%)	0.2
Treatment concluded by mutual agreement	12 (21%)	4 (7%)	0.02	13 (22%)	4 (7%)	0.01
Continued with psychotropics only	6 (10%)	7 (12%)	0.8	6 (11%)	8 (14%)	0.6
Person received treatment as desired^a^ (mean (SD))	8.2 (1.7)	7.6 (1.9)	0.06	8.1 (1.7)	7.6 (1.8)	0.09
Workers able to work as desired^a^ (mean SD))	8.2 (1.4)	7.5 (1.9)	0.01	8.2 (1.5)	7.5 (1.8)	0.01

^a^0=strongly disagree, 10 = strongly agree

*SD* Standard deviation

During the follow-up period from recruitment to the year 2024, a greater proportion of service users in the research group had their treatment conclude through mutual agreement. The proportion of service users transitioned to maintenance treatment (psychotropics only) did not differ significantly between the groups.

After weighting for baseline characteristics, treatment in the research group was associated with significantly higher odds of ending through joint agreement, with an odds ratio of 3.8 (95% CI: 1.2–13.0, *p* = 0.026).

## Discussion

### Overview of Main Findings

This naturalistic within-region comparison conducted within the Western Lapland catchment area examined whether long-standing organizational differences between the Open Dialogue–based hospital district units and the municipally governed outpatient unit—which all originally operated as Open Dialogue services—were reflected in treatment practices, service experiences, and outcomes. The organizational split—where one outpatient unit operated for more than two decades under separate municipal governance while the others remained within the hospital district—created a unique opportunity to explore how variations in governance, training structures, and service organization may have shaped dialogical practices and, in turn, the experiences and outcomes of service users and staff.

Across all units, service users reported generally rapid access, continuity, and overall satisfaction. However, consistent differences emerged between the hospital district units and the municipal unit, suggesting that treatment cultures and practices have diverged over time despite their shared historical roots in Open Dialogue.

### Treatment Characteristics and Experiences

Across the hospital district units, several practices commonly associated with Open Dialogue —such as continuity of care, involvement of family and social networks, home visits, and staff with formal Open Dialogue training—were more frequently reported. Although the study design does not allow attribution of these differences to specific organizational features, the pattern suggests that units that remained within the same administrative structure were better able to maintain a more consistent dialogical orientation. The differences in service experiences parallel the observed variations in treatment content. In particular, family and network meetings were rated as more helpful in the hospital district group, indicating that the same intervention may have been delivered differently across organizational contexts.

### Interpreting Outcome Patterns

Service users treated in hospital district units reported somewhat greater improvement and were more likely to conclude treatment through mutual agreement in follow-up. These findings must be interpreted cautiously given the post hoc, non-randomized design in which causal inferences cannot be drawn. Nevertheless, because intake procedures and many structural features were similar across units at the time of the survey, the pattern is consistent with the interpretation that a more coherent dialogical culture—reflected in treatment characteristics and experiences—may have supported more positive service experiences and outcomes.

However, when compared with previous studies on adolescent (Bergström et al., 2022) and psychosis cohorts (Bergström et al., 2018) from the same catchment area, several differences were observed. First of all, nearly all participants in the present sample had a formal psychiatric diagnosis, likely reflecting national policy changes that require a recorded diagnosis for access to services. Changes in staffing—including the retirement of clinicians who had been central to the early development of Open Dialogue—may also have influenced treatment content, such as the increased use of psychotropic medication and the longer treatment durations observed here relative to earlier Open Dialogue studies (Bergström et al., 2022; 2018). There was some indication in earlier PSILEAPS analyses that the study’s recruitment procedures tended to underrepresent brief or crisis-based contacts (Bergström et al., 2025). Such selection effects may have resulted in a sample skewed toward longer-term service users, partially explaining differences from previous cohorts derived from population registers, which were not subject to similar forms of sample loss.

### Organizational Factors Potentially Shaping Differences

Several systemic features may help explain the observed variation between clinics. According to the results, the hospital district units had overall lower staff turnover, more extensive Open Dialogue training, and a stable, threshold-free service structure. In contrast, the Kemi outpatient clinic had operated for decades under a municipal governance model characterized by reimbursement-driven service boundaries and administrative requirements to direct care locally. Although the Keropudas outpatient clinic provided centralized intake for the region, coordination of care have been more straightforward with the co-located Tornio unit compared to municipal services, which often required referral back to local providers. This likely increased efficiency and supported smoother care pathways, whereas in Kemi the more fragmented structure was associated with a lower proportion of treatment episodes ending through mutual agreement. This pattern may have contributed to longer treatment durations and, over time, larger cumulative caseloads—potentially reinforcing a cycle in which extended episodes further constrain flexibility and increase the likelihood of continued long-term contact.

Even though no strict linear causal inference can be made, previous studies suggest that such structural constraints may limit the ability to sustain dialogical practices at scale and contribute to variability in how core elements are implemented. Fragmented service systems, reimbursement logics, and unstable funding frameworks have repeatedly been shown to constrain fidelity to OD’s structural principles, resulting in only partial or “OD-inspired” implementations (Buus et al., 2021; Heumann et al., 2023; Waters et al., 2021). Organizational factors—such as service integration, continuity of teams, and the presence of a clear, shared OD framework—have likewise been central to the successful adoption and long-term maintenance of dialogical practices in other contexts (Buus et al., 2021; Lennon et al., 2023). Moreover, prior studies emphasize that OD implementation depends not only on formal structures but also on leadership style, local culture, and staff attitudes: adaptive, dialogically oriented leadership and a supportive team culture appear to buffer uncertainty and resistance, whereas high staff turnover, ambiguous mandates, and strong professional hierarchies tend to erode OD practices over time (Skourteli et al., 2023; Heumann et al., 2023; Lennon et al., 2023). In this light, the more coherent, hospital-district–based organizational context may have provided stronger conditions for maintaining OD treatment culture than the municipally governed Kemi clinic.

### Strengths and Limitations

This study has several strengths. First, it draws on real-world data from a region with a well-documented history of Open Dialogue service development. This context provides a rare opportunity to examine how treatment cultures evolve under different organizational structures and how such variations influence clinical practice and user experiences. Additionally, the study contributes to ongoing discussions about the sustainability of dialogical practices and highlights the structural and cultural conditions important for their long-term maintenance.

However, several limitations must be acknowledged. First, this was a post hoc analysis of data originally collected for a different primary purpose, which inherently limits the degree of control over variable selection, measurement procedures, and data completeness. As such, some potentially relevant factors could not be examined or were not available in the level of detail that a prospectively designed comparative study would allow. Second, the questionnaires were signed to enable linkage to the corresponding treatment process. Although participants had the opportunity to complete the forms privately and return them in sealed envelopes, the non-anonymous format may have influenced how some individuals chose to respond. This potential response bias is acknowledged as a limitation.

Thirdly, previous analyses (Bergström et al., 2025) suggest that the sample may have disproportionately included individuals with longer treatment histories or those already engaged with services, limiting generalizability. However, the attrition of participants receiving brief, crisis-oriented care—most typical of the OD mandate—was observed primarily in the Keropudas outpatient clinic, which also treated more severe cases overall. Since this pattern would be expected to bias outcomes *against* the hospital-district units rather than in their favor, the higher rate of e.g. mutually agreed treatment conclusions in these units are unlikely to be explained by selection bias.

While we successfully adjusted for key demographic and clinical differences, unmeasured confounding factors likely remain, and it is not possible to isolate specific factors that contributed to the differences between units. For example, beyond variations in OD treatment culture and training, differences in cumulative caseloads and local service infrastructure may have served as mediating factors. Moreover, although both groups were drawn from the same geographic region and from units with a similar staffing and patient profile, sociocultural differences—such as the greater socioeconomic burden in Kemi—may have influenced outcomes. That said, according to results more severe cases were treated particularly at the Keropudas outpatient clinic, and despite this, the results remained consistent in the same direction.

Another limitation is the limited statistical power, which did not reach the level initially planned (Bergström et al., 2022b); the sample size was insufficient to detect all potential between-group differences with confidence, and several estimates should therefore be interpreted cautiously. In particular, p-values may be unstable in small samples and should not be taken as definitive evidence for the presence or absence of an effect. The possibility of both type I and type II errors remains, and the direction and magnitude of differences may be more informative than strict significance thresholds. Nonetheless, the study successfully achieved its primary aim by offering new insights that can inform future research and hypothesis generation related to dialogical practices.

## Conclusion

This naturalistic within-region comparison examined how long-standing differences in service organization are reflected in treatment practices and perceived outcomes. Although all units originally developed within the same Open Dialogue (OD) service system, the hospital-district units—where OD training, supervision, and team continuity had been more consistently sustained—were more likely to offer treatment elements central to dialogical work, such as home-based family and network meetings facilitated by OD-trained staff. These practices were also rated as more helpful by both service users and staff, suggesting not only greater frequency but qualitative differences in how they were delivered.

Treatment in the hospital-district units was also associated with somewhat greater self-reported improvement and a higher likelihood that treatment ended through mutual agreement between service users and staff. While the post hoc, non-randomized design, potential selection bias, and modest sample size limit causal interpretation, the findings offer hypothesis-generating evidence that organizational structure shape how dialogical practices are sustained and experienced in routine care.

Taken together, the findings from this and previous studies suggest that the practice referred to as Open Dialogue—when associated with more favorable outcomes—is not merely a clinical method but an orientation and treatment culture that depends on the wider ecology of the service system. Fragmentation, unstable funding, high staff turnover, and limited training opportunities appear to narrow the scope of dialogical work and are associated with longer treatment episodes and less helpful experiences of social network involvement. In this sense, the present study supports the view that sustaining dialogical practice requires more than the adoption of specific techniques; it calls for a broader epistemic, strategic and structural transformation of mental health services—aligned with the World Health Organization’s (2021) call for holistic, rights-based reform rather than piecemeal model implementation.

## Data Availability

Due to Finnish legislation on the secondary use of healthcare data and the associated risk of identifiability, the full dataset cannot be shared. Upon reasonable request, parts of the anonymized dataset may be obtained from the first author.

## References

[CR1] Alanen, Y. O. (2009). Towards a more humanistic psychiatry: Development of need-adapted treatment of schizophrenia group psychoses. *Psychosis*, *1*(2), 156–166.

[CR2] Andersen, T. (1987). The reflecting team: Dialogue and meta-dialogue in clinical work. *Family Process,**26*(4), 415–428. 3691768 10.1111/j.1545-5300.1987.00415.x

[CR3] Anderson, H., & Goolishian, H. A. (1988). Human systems as linguistic systems: Preliminary and evolving ideas about the implications for clinical theory. *Family Process,**27*, 371–393. 3234525 10.1111/j.1545-5300.1988.00371.x

[CR4] Austin, P. C., & Stuart, E. A. (2015). Moving towards best practice when using inverse probability of treatment weighting (IPTW) using the propensity score to estimate causal treatment effects in observational studies. *Statistics in Medicine,**34*(28), 3661–3679. 10.1002/sim.6607 26238958 10.1002/sim.6607 PMC4626409

[CR5] Bergström, T., Seikkula, J., Alakare, B., Mäki, P., Köngäs-Saviaro, P., Taskila, J. J., Tolvanen, A., & Aaltonen, J. (2018). The family-oriented open dialogue approach in the treatment of first-episode psychosis: Nineteen-year outcomes. *Psychiatry Research,**270*, 168–175. 10.1016/j.psychres.2018.09.039 30253321 10.1016/j.psychres.2018.09.039

[CR6] Bergström, T., Seikkula, J., Alakare, B., Kurtti, M., Köngäs-Saviaro, P., Löhönen, E., Miettunen, Jouko, Mäkiollitervo, Hannele, Taskila, Jyri J.., Virta, Katriina, & Valtanen, K. (2022a). The 10-year treatment outcome of open dialogue-based psychiatric services for adolescents: A nationwide longitudinal register-based study. *Early Intervention in Psychiatry,**16*(12), 1368–1375. 10.1111/eip.13286 35332989 10.1111/eip.13286 PMC10078679

[CR7] Bergström, T., Biro, M., Gauffin, T., Haaraniemi, T., Kurtti, M., Köngäs-Saviaro, P., Hietasaari, T., Holma, J., Jääskeläinen, E., Laitila, A., Löhönen, E., Miettunen, J., Nikanne, P., Petäjäniemi, J., Seikkula, J., Tarkka, M., Taskila, J., & Mäkiollitervo, H. (2022b). Protocol for a participatory survey to investigate the long-term effectiveness of adult psychiatric services (PSILEAPS): A prospective exploratory cohort study. *Psychiatria Fennica,**53*, 220–229.

[CR8] Bergström, T., Gauffin, T., Hietasaari, T., Koivuniemi, N., Leinonen, A., Öfverberg, E., Viitakoski, R., Haaraniemi, T., Partanen, K., Rantamaa, K., Yrjänheikki, V., Jauhiainen, T., Olli, A., Miettunen, J., & Petäjäniemi, J. (2025). A participatory survey to investigate the long-term effectiveness of adult psychiatric services (PSILEAPS): Baseline data and recruitment experiences. *Community Mental Health Journal*. 10.1007/s10597-025-01462-z 40067545 10.1007/s10597-025-01462-z PMC12408686

[CR9] Buus, N., Ong, B., Einboden, R., Lennon, E., Mikes-Liu, K., Mayers, S., & McCloughen, A. (2021). Implementing open dialogue approaches: A scoping review. *Family Process*, *60*(4), 1117–1133. 10.1111/famp.12695 34322874 10.1111/famp.12695

[CR10] Haarakangas, K. (1997). *). The voices in treatment meeting: A dialogical analysis of the treatment meeting conversations in family–centred psychiatric treatment process in regard to the team activity*. University of Jyväskylä. Jyväskylä Studies in Education, Psychology and Social Research 129.

[CR11] Heumann, K., Kuhlmann, M., Böning, M., Tülsner, H., Pocobello, R., Ignatyev, Y., Aderhold, V., & von Peter, S. (2023). Implementation of open dialogue in Germany: Efforts, challenges, and obstacles. *Frontiers in Psychology,**13*, Article 1072719. 10.3389/fpsyg.2022.10727 36846479 10.3389/fpsyg.2022.1072719 PMC9948650

[CR12] Lennon, E., Hopkins, L., Einboden, R., McCloughen, A., Dawson, L., & Buus, N. (2023). Organizational change in complex systems: Organizational and leadership factors in the introduction of open dialogue to mental health care services. *Community Mental Health Journal*, *59*(1), 95–104. 10.1007/s10597-022-00984-0 35585467 10.1007/s10597-022-00984-0 PMC9813114

[CR13] Overall, J. E., & Gorham, D. R. (1962). The Brief Psychiatric Rating Scale. *Psychological Reports,**10*, 799–812. 10.2466/pr0.1962.10.3.799

[CR14] Pocobello, R., Camilli, F., Alvarez-Monjaras, M., Bergström, T., von Peter, S., Hopfenbeck, M., Aderhold, V., Pilling, S., Seikkula, J., & El Sehity, T. J. (2023). Open dialogue services around the world: A scoping survey exploring organizational characteristics in the implementation of the Open Dialogue approach in mental health services. *Frontiers in Psychology,**14*, Article 1241936. 10.3389/fpsyg.2023.1241936 38023059 10.3389/fpsyg.2023.1241936 PMC10668593

[CR15] Razzaque, R., & Stockmann, T. (2016). An introduction to peer-supported open dialogue in mental healthcare. *BJPsych Advances*, *22*(5), 348–356.

[CR16] Seikkula, J., & Olson, M. E. (2003). The open dialogue approach to acute psychosis: Its poetics and micropolitics. *Family Process*, *42*(3), 403–418. 14606203 10.1111/j.1545-5300.2003.00403.x

[CR17] Seikkula, J., Alakare, B., Aaltonen, J., Holma, J. M., Rasikangas, A., & Lehtinen, V. (2003). Open dialogue approach: Treatment principles and preliminary results of a two-year follow-up on first episode schizophrenia. *Ethical Human Psychology and Psychiatry*, *5*(3), 163–182.

[CR18] Seikkula, J., Aaltonen, J., Alakare, B., Haarakangas, K., Keränen, J., & Lehtinen, K. (2006). Five-year experience of first-episode nonaffective psychosis in open dialogue approach: Treatment principles, follow-up outcomes, and two case studies. *Psychotherapy Research*, *16*(2), 214–218. 10.1080/10503300500268490

[CR19] Seikkula, J., Alakare, B., & Aaltonen, J. (2011). The comprehensive OpenDialogue approach in Western Lapland: II. Long-term stability of acute psychosis outcomes in advanced community care. *Psychosis,**3*(3), 192–204.

[CR20] Skourteli, M. C., Issari, P., Dimou, L., Antonopoulou, A. O. A., Bairami, G., Stefanidou, A., Kouroglou, V., & Stylianidis, S. (2023). The introduction and implementation of open dialogue in a day center in Athens, Greece: Experiences and reflections of mental health professionals. *Frontiers in Psychology,**14*, Article 1074203. 10.3389/fpsyg.2023.1074203 37303906 10.3389/fpsyg.2023.1074203 PMC10249676

[CR21] Waters, E., Ong, B., Mikes-Liu, K., McCloughen, A., Rosen, A., Mayers, S., Sidis, A., Dawson, L., & Buus, N. (2021). Open dialogue, need-adapted mental health care, and implementation fidelity: A discussion paper. *International Journal of Mental Health Nursing,**30*(3), 811–816. 10.1111/inm.12866 33848029 10.1111/inm.12866

[CR22] World Health Organization. (2021). *Guidance on community mental health services: Promoting person-centred and rights-based approaches*. World Health Organization.

